# Rheological and Mechanical Properties of Resin-Based Materials Applied in Dental Restorations

**DOI:** 10.3390/polym13172975

**Published:** 2021-09-01

**Authors:** Xinyuan Zhang, Qi Zhang, Xin Meng, Yuting Ye, Daoshuo Feng, Jing Xue, Hanbing Wang, Haofei Huang, Ming Wang, Jing Wang

**Affiliations:** School of Chemistry and Chemical Engineering, Shandong University of Technology, 266 Xincun Rd., Zibo 255000, China; xinyuanzhangsdut@163.com (X.Z.); qizhangsdut@163.com (Q.Z.); mengxinsdut@163.com (X.M.); yutingyesdut@163.com (Y.Y.); fengdaoshuosdut@163.com (D.F.); xuejingsdut@163.com (J.X.); wanghanbing0119@126.com (H.W.); 1982hhf@163.com (H.H.); wangmingmw@sdut.edu.cn (M.W.)

**Keywords:** dental restorative materials, rheology, dental resin composite, dental bonding agent, resin luting cement

## Abstract

Resin-based materials have been prevalent for dental restorations over the past few decades and have been widely used for a variety of direct and indirect procedures. Typically, resin-based dental materials are required to be flowable or moldable before setting and can provide adequate mechanical strength after setting. The setting method may include, but is not limited to, light-curing, self-curing or heating. In this review, based on different indications of resin-based dental materials (e.g., dental filling composite, dental bonding agent, resin luting cement), their rheological and mechanical properties were reviewed. Viscous and flexible properties were focused on for materials before setting, while elastic properties and mechanical strength were focused on for materials after setting. At the same time, the factors that may affect their rheological and mechanical properties were discussed. It is anticipated that the insightful information and prospections of this study will be useful to the future development and fabrication of resin-based dental restorative materials.

## 1. Introduction

Oral diseases are prevalent and affect human health from many aspects, and dental caries can lead to many adverse consequences as one of the most common stomatological problems [1]. As a tissue with weak metabolism, dental hard tissue cannot recover its from defects through cell regeneration once it is destroyed. Therefore, dental restorations are necessary to recover their normal shape and functions.

In order to mimic the appearance and functions of original human teeth, dental restorative materials have to meet certain requirements. The physical properties of dental restorative material are expected to be the same as dental tissue, the wear resistance is expected to be similar to enamel, and they are also expected to be easily and fully combined with the dental tissue to be restored [2]. Optical properties are favorable and biological compatibility and tasteless properties are expected when the materials are applied to the organisms [2]. Color matching and color stability are also required [3,4]. In addition to the final result, dental filling materials should also be easy to handle and have good adhesion to the teeth with little shrinkage [5], which is more likely to provide a strong chemical bond so that the filled teeth are durable and stable [6]. Resin-based dental restorative materials are a class of composites used for direct or indirect repair with polymerizable resin as a matrix and inorganic filler or fiber as reinforcement [4]. They are typically formulated with methacrylate monomers, initiators and fillers and are cured through radical polymerizations, resulting in hard materials. The technique of resin-based dental restorative materials is replacing many metal-based restorative materials in recent years [7]. The most prominent advantage of resin-based dental restorative materials is that they can match the appropriate color of the teeth in appearance and form a bond retention with the tooth tissue.

**Figure 1:**
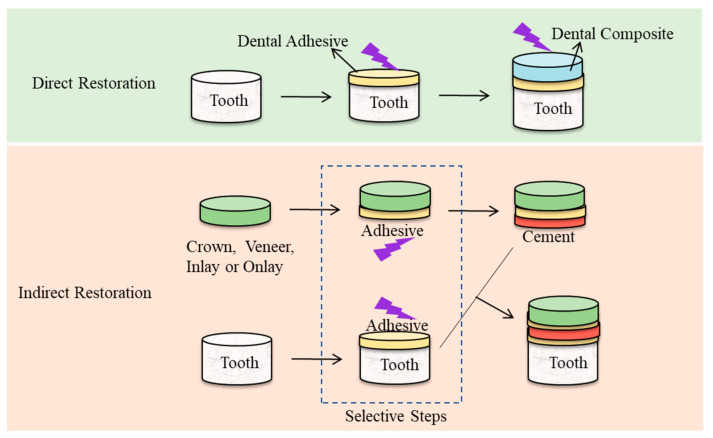
Resin-based materials applied in direct and indirect restorations.

Resin-based materials have been widely used in direct and indirect restorations (Figure 1), where dental composite and dental adhesives are mainly used in direct restorations, while resin luting cement is applied in indirect restorations. Dental resin composites have been widely used in commercial production since the 1960s because of their excellent mechanical and aesthetic properties [8]. Along with their development, people have been working to optimize their performances, but they still have some defects that cannot be ignored such as polymerization shrinkage and defective physical and mechanical properties [8]. Resin-based composites have undergone many modifications as they evolved and represent the most relevant restorative materials in dental practice today [9]. In this review, the rheological and mechanical properties of resin-based restorative materials with different indications (e.g., dental filling composite, dental adhesive and resin luting cement) are discussed, and the cutting-edge techniques, challenges and future development of resin-based dental restorative materials are also covered.

## 2. Dental Filling Composite

Dental filling material is mainly used for dental cavity filling. The common materials are silver amalgam, composite resin and glass ion complex [10,11]. In clinics, dentist of-ten chooses different materials to fill the cavity caused by dental caries according to the location of the cavity, the size of the bite force and the patient’s health and aesthetic requirements [12]. Composite resins are preferred in recent years because of their greater tensile strength and less shrinkage and deformation during curing [13,14]. In the past, composite resins were softer than silver amalgam and were mainly used for filling cavities in anterior teeth. However, the mechanical properties and wear resistance of composite resins that have emerged recently have been greatly improved, and they are also widely used in the filling of posterior teeth and large-area cavities [15].

Composite resin is mainly composed of resin matrix, inorganic filler, trigger system and other trace additives, and the types and content of each component vary according to the material [16]. The resin matrix (typically 15–50% weight percent) is the main component of the polymerizable part of composite resin to ascribe the mixed compositions with plasticity and curing properties, which is also the main component that determines the physical mechanical properties [17]. The most commonly used components of resin substrates are bisphenol A glycidyl methacrylate (Bis-GMA), modified Bis-GMA, triethylene glycol dimethacrylate (TEGDMA) and other monomers [18]. Because the viscosities of these monomers are very large and cannot be mixed into a sufficient amount of inorganic fillers, it is difficult to obtain the desired enhancement effect and plasticity. Part of diluted monomer is usually added to form a resin matrix in order to meet the requirements [19]. Resin matrix combines other components such as inorganic fillers to form plastic pastes, thus giving the material required handling properties. The fluidity of the composite resin not only determines its handling property but also affects its fit to the prepared dental surface and the formation of prosthesis [20]. Dental composites can be divided into many types for different characteristics (e.g., filler sizes, curing method, indications) and universal versus flowable based on their consistency is the most commonly used distinguishing way for commercial products designed for different clinical applications [20]. In this case, universal dental composites and flowable dental composites are categorized in this review.

### 2.1. Universal Dental Composite

A universal dental composite is designed to restore carious lesions or structural defects in teeth for nearly all conditions. Since the 1970s, photocurable dental composite resin materials have been widely used to repair caries and other damage fillers due to their excellent materialization properties and aesthetic clinical medical properties compared to silver amalgams [21]. Some composite filling resins contain very high inorganic filler content leading to difficulty in extruding it from the surrounding [22], so they are often used for the repair of large defects in the posterior teeth [15]. In dentistry, the preparation process for composite resins is complex, so some filling composite resins must be extruded, which requires uncoagulated composite materials having great variability [23]. Over the past decade, improvements have been made to the non-stick and moldable properties of dental composites to make them more suitable for dental fillings [24].

#### 2.1.1. Rheological Properties before Curing

The ideal rheological properties of universal dental composites may include low fluidity when extruding force is applied and fixed geometry when the extruding force disappears [23]. To achieve this effect, it is usually necessary to increase the viscosity of the material and reduce the adhesion of the material, where low adhesion is very important for the smooth placement of material from the packaging container into the prepared nest hole. Watts et al. [25] studied two commercial resin substrates within the shear rate range by capillary tube technology in 1980, and they observed that the system appears to be pseudoplastic for the component and slightly pseudoplastic to Newtonian after mixing. Al-Sharaa et al. [23] evaluated the stickiness of composite resins prior to setting and their fluidity through laboratory viscosity measurements. Twelve different resin-based composites were selected according to the composition and fill quantity of the resin matrix, and they found that the viscosity of resin-based composites may vary depending on the filling volume of inorganic fillers, the shape and size of particles and the organic composition of solids, and the increased viscosity is correlated to decreased stickiness [23]. Tawas et al. [26] studied the fluency characteristics of unfilled resins using a cone-plate viscometer. When the Bis-GMA monomer and several mixtures were studied in different shear rate ranges, it was found that their rheological properties were different, and the apparent viscosity of the resin increases when the shear rate is increased. When the resin is completely filled with Bis-GMA, the viscosity of the composite material was 426.6 N·s/m^2^ at 24.1 °C. The addition of a methyl methacrylate (MMA) monomer diluent can significantly decrease the viscosity. The viscosities of commonly used monomers and their combinations are listed in Table 1, and there is a wide selection of diluent monomers in different circumstances. Al-Ahdal et al. [27] used a parallel-plate rheometer to study 14 commercial composite resins with different resin matrices, and they found a strong temperature dependence of viscosity which can be explained by the Arrhenius formula. The viscosity ranged from 50 Pa·s to 349.33 kPa·s when the temperature was at 25 °C, while it decreased to 30 Pa·s to 132 kPa·s when the temperature increased to 37 °C.

Inorganic fillers and resin substrates are two distinct substances, and their mechanical properties are also very different. When the non-surface-treated inorganic fillers and resin matrix are mixed, the mechanical performance of the resulting composite resin is poor, leading to a lack of bonding between the filler and the resin matrix [31]. In order to improve the binding force between the filler and the resin, a coupling agent is commonly used for surface modifications of the filler [32]. In the resin matrix of dental composites, various inorganic particles with different compositions are used, such as silica, alumina, silicate glass, quartz and ceramics [31,33]. The filling phase is incorporated into the organic matrix of the dental composite to customize different mechanical properties with the purpose of imitating the properties of the dental tissue they replace [34]. Research by Lee et al. [28] showed that as the volume fraction of barium glass filler increases, the viscosity of the composite material increases significantly. For the same mass fraction filler, using 0.04 μm silica instead of 0.7 μm barium glass filler can greatly increase the composite material of viscosity from 12.9 to 61.1 Pa·s. The storage modulus of universal composites differs by different products; Charisma (Kulzer, Germany) has a storage modulus of 164.4 Pa before cure, which is much lower than Cearfil (Kuraray, Japan) with a storage modulus of 1006.9 Pa designed for similar clinical indications [28]. 

#### 2.1.2. Curing Properties

The curing reaction of the composite material with methacrylates as the resin matrix is a polymerization reaction initiated by active free radicals [35]. The curing process of the bulk filling resin is also driven by light or chemistry to generate free radicals by the photoinitiators becoming the active center. This promotes the conversion of C=C double bonds in monomer molecules into C–C single bonds to form a polymer chain, which in turn presents a polymer network structure. The visible light curing trigger system consists of photosensitive agents and promoters. The common photosensitizers are camphorquinone (CQ) [36] and benzophenone (BP) [37]. Zorzin et al. [38] used a 5 mm diameter polymethyl methacrylate (PMMA) rod as a bonding matrix in a universal testing machine to study the polymerization shrinkage of composite materials. The research results have shown that extending the curing time will increase the polymerization shrinkage rate of most composite materials by 0.06 to 0.2 MPa. Sun et al. [39] used X-ray microcomputer tomography to determine the volume change of composite materials containing different mass fractions of Bis-GMA and TEGDMA after polymerization shrinkage. The experimental results have shown that when the mass ratio of Bis-GMA: TEGDMA is 7:3, the polymerization shrinkage of the composite material is 60%; when the mass ratio of Bis-GMA: TEGDMA is 3:7, the polymerization shrinkage of the composite material is 90%. It can be seen that the more Bis-GMA, the lower the polymerization shrinkage ability of the composite material.

Fujita et al. [40] used resin composite pastes containing particles of different diameters to study the effect of silicon filler particle diameter on the polymerization conversion of resin composite materials. They found that fillers with larger diameters might hinder the transmittance of curing light, resulting in smaller polymerization conversion rates of the composite material, so smaller particle sizes were recommended. Zorzin et al. [41] used FTIR-ATR-spectroscopy to measure the degree of conversion of four commercial bulk-filled composite materials at different curing depths. When the curing time specified by the manufacturer exceeded 10 s, the degree of conversion of the composite resin decreased. The reason for the decrease in the degree of conversion was considered as the decreased transparency of the filler after prolonging the curing time, so reducing the filler–matrix ratio can increase the polymerization conversion rate. D’Alpino et al. [42] found that the nano-filled composites have a higher degree of conversion than particle-filled composites, and the well dispersion of fillers producing composites with better consistency was considered as an important reason. 

The depth of cure is another critical parameter of dental composites and relates to the curing efficacy through the depth of composites after the curing process. Properly prolonging the irradiation time, the curing depth will also increase, but after the irradiation time exceeds 60 s, the increase in the curing depth becomes insignificant [43]. The curing depth is closely related to the effective wavelength and light intensity of the curing lamp, and the higher the light intensity, the deeper the curing [43]. The relevant standards [44] stipulate that the curing time of chemical curing composite resin is not greater than 5 min at room temperature and not less than 90 s. The relevant standards [44] stipulate that the curing depth of the 20-s composite resin should not be less than 1.5 mm, and the curing depth of most photo-curable composite resins should be 2.0 to 3.0 mm. The experimental study by Fujita et al. [40] showed that when the filler diameter of the composite material increased from 0.05 μm to 2.0 μm, the transmitted amount of the light decreased from 68.6% to 9.7%, and the curing depth decreased from 14.7 mm to 7.0 mm. Therefore, reducing the filler diameter of the composite material can make the curing depth of the composite deeper. Dunne et al. [45] found that the curing depth of light is related to the separation distance and light output intensity. For composite materials, the greater the distance between the light source and the composite material, the smaller the curing depth. Increasing the light output intensity should appropriately reduce the curing time. When increasing the curing time from 20 s to 60 s, the curing depth can be increased by about 1.4 times.

#### 2.1.3. Mechanical Properties after Curing

**Figure 2:**
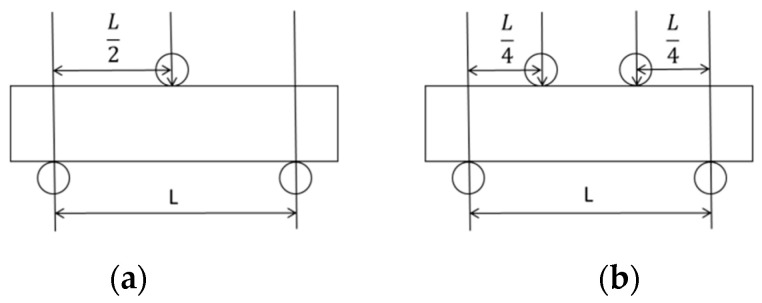
Schemes of (**a**) three-point bending test; (**b**) four-point bending test. Schemes were adapted from reference [47].Reproduced with permission from Chitchumnong, P.; Brooks, S.C.; Stafford, G.D.; Dental Materials; published by Elsevier, 1989.

Composite resin can be used to repair nearly all kinds of tooth defects which should have a certain mechanical strength to resist the oral function of various stresses without damage to shedding. After curing, these composite filling resins have increased hardness and wear resistance, which can better withstand chewing forces. Mechanical properties, including compressive strength, bending strength and elastic modulus, are the main experimental basis for selecting resin materials. Mechanical strength can be tested by three-point bending or four-point bending (Figure 2) [46,47]. Both test methods can be performed in the same way and can be performed on samples of the same size [47].

Yao et al. [48] used the three-point bending method to characterize the bending characteristics of interpenetrating phase composite (IPCs), and the thinner fiber helps to improve the bending strength. When the diameter of the fiber is reduced from 160 μm to 90 μm, the bending strength increases from 76 MPa to 98 MPa. When Chitchumnong et al. [47] used three-point bending and four-point bending to measure heat-polymerized industrial nylon and auto-polymerized materials, they found that, for the same material, the flexural strength measured by three-point bending is always higher than that measured by four-point bending. However, after correcting with the correction formula, the actual values measured by the two methods are relatively close. Ikejima I et al. [49] have shown that within a certain range (≤10%), the bending strength of the composite resin is proportional to the content of the filler. The bending strength of the composite resin increases with the content of the filler, but the bending strength does not increase, or even decrease, when a certain limit is exceeded. Cho et al. [50] compared the compressive strength of eight materials and found that the compressive strength varies greatly between different types of materials. The theoretical photocurable composite resin compressive strength should be 246–448 MPa, and the theoretical self-curing titanium composite resin pressure strength should be 212–280 MPa, but there is still a gap between the experimental data and the theoretical data. Therefore, the testing process should be longitudinal to controlled clinical trials of the material. Chadwick et al. [51] put the composite resin in distilled water at different temperatures and then conducted thermal cycling. They found that for different composite resins, after the temperature changes, their compressive strengths also changed. For composite resin materials, their compressive performance is generally 240 to 320 MPa. Carreiro et al. [52] also conducted a similar experiment. They put different types of composite resin materials in distilled water for 180 days and then took them out to test their compressive strengths. They found that the compressive performance of the same composite material did not change significantly. The difference in compressive strength between different types of composite materials may be related to filler volume and resin formulation.

### 2.2. Flowable Dental Composite

In recent years, manufacturers have reduced viscosity by changing the filler content while using different resin matrix monomers, which has allowed for composite resins to better adapt to cavity preparation. This ensures that the resin can flow effectively into and adapt to the prepared nest when filling when the material is extruded from a compression device, while the material have no resistance to the extrusion force [53]. Flowable composite is a new type of filling material that has become popular in recent years, which can be used as liners or bases combined with universal composite [54]. It has the characteristics of being easy to handle, good permeability and fluidity, which can penetrate into the micro hole and tooth edge well, producing good adhesion with less shrinkage than ordinary composite materials [55]. In addition to the restorative purposes, flowable dental composites can also be used for pit and fissure sealing, porcelain repair and small core build up owing to its flowability [55].

#### 2.2.1. Rheological Properties before Curing

Better fluidity is a distinct characteristic of flowable composite compared with universal composite. Flowable resin composites represent low-viscosity resin composites resulting from filler contents 37–53% lower (volume) than conventional composites [53]. The content of fluidity varies significantly from one product to another. Hence, the viscosity and flow characteristics of flowable resin composites can have a potential influence on their handling properties and thus on their clinical indications. As a result of differences in viscosity, flowable composites vary considerably in polymerization shrinkage, stiffness and other physical properties [55].

Ergucu et al. [56] studied three representative materials of flowable, orthodontic and reparative composites and found that significant differences existed in the flow parameters. Especially, medium viscosity orthodontic composites have a greater spillage tendency compared with low viscosity flow composites [57]. A study [58] shows that the higher the content of visible light curing composite filler, the worse its dimensional stability. Filler content and resin matrix properties are two factors that affect the mechanical properties of fluid composites [59]. Beau et al. [60] compared the viscosity of eight flowable resin composites. The instant viscosity of flowable composites ranged from 141.53 Pa s to 30,286 Pa s, which exhibited an increase trend with the increase of testing time. The incorporation of fillers may increase their viscosities, but there was not a confirmed correlation found between the rheological properties and the filler parameters in their study. Jager et al. [61] also found that filler content may be an important parameter for the fluidity of flowable resin composites, but there was not a quantitative method to describe their relationships. Typically, the higher of the filler content, the greater the viscosity of the flowable composite material [62].

#### 2.2.2. Curing Properties

Due to the refraction and scattering of the opaque material in the resin, at a certain depth, the ratio of the resin hardness to the surface layer hardness of 0.8 or more is considered to be fully cured [43,63]. The curing depth of the flowable composite material can reach 4 mm [44], and the curing depth of 4–6 mm at a time is the most prominent advantage of the bulk filling resin.

The clinical success of resin composite repair is inseparable from parameters such as curing depth, polymerization shrinkage rate and degree of conversion [64]. There typically exists a difference in the depth of cure and the degree of conversion between different products. Maciel et al. [65] prepared a series of flowable composites with different concentrations of CQ (ranging from 0.25% to 2% by weight), and the obtained DC% increased with the increase of CQ concentration (range from 31.01% to 46.46% in their study). When coupled with another co-initiator, dimethylamino ethyl methacrylate (DMAEMA), the DC% can be slightly improved. The volume shrinkage of flowable composite is typically greater than that of universal composite [66], so more dedication is needed in this topic. He et al. [67] synthesized a new photo-curable monomer methyl dimethylformate ester amine and added it to benzene-GMA/TEGDMA to reduce polymerization shrinkage. The resulting DC% of prepared flowable composite can reach 60%, and the polymerization shrinkage stress can be reduced by approximately 80% (from −5 MPa to −0.8 MPa) without compromising the physical properties and wear of the resin composites. Aung et al. [68] found that the adequacy of polymerization is mainly dependent on the composition of the resin composite. Although composite restorations are widely used in dental clinics, the polymerization shrinkage is still a big concern, leading to clinical failure and adverse consequences [69].

#### 2.2.3. Mechanical Properties after Curing

The mechanical properties of flowable composites after curing are similar to those of universal composites. Tjandrawinata et al. [70] evaluated the flexural strength and shear bond strength of flowable composites, and they found that the flexural strength of tested flowable composite was about 110–130 MPa, while the shear bond strength to enamel was about 15 to 25 MPa. Filler is an important factor that can significantly affect the mechanical strength of composite materials [4]. In another study by Mirica et al. [71], it was reported that the flexural strength can be improved to 60–90 MPa with the addition of around 50% appropriate fillers. At the same study [71], the prepared flowable composite had compressive strength of 182–310 MPa and flexural modules of 2.34–6.32 GPa, which was higher than those of the conventional flowable composites owing to the selection of functional fillers. Maciel et al. [65] has also found that the photoinitiator concentration may affect the conversion degree and mechanical properties of flowable composites.

Since flowable composite has much lower viscosity than universal composite, which can be formulated as similar as dental adhesives, self-adhesive flowable composites are proposed to simplify clinical procedures. In this way, the step of applying dental adhesive between the prepared dental surface and dental composite can be eliminated. Asiri et al. [72] compared the shear bond strength of flowable composite with dentin and a conventional adhesive, and they found that the flowable composite has lower shear bond strength compared to a conventional adhesive-restored dentin. David et al. [73] found that the adhesive strength of self-adhesive flowable composite to dentin and enamel is lower than that of conventional bonding with composite resins and self-etching adhesives. Tuloglu et al. [74] also found that the adhesive strength of self-adhesive flowable composites is lower than that of teeth repaired with traditional adhesive. Therefore, it was suggested that preventive measures should be taken for the selection of self-adhesive flowable composite resins until their adhesion stability to tooth tissues and long-term clinical properties are evaluated [73].

## 3. Dental Bonding Agent

Dental bonding agent (or dental adhesive) is a component used to bond a prosthesis or prosthetic material to the surface of hard and soft tissues in the oral cavity [75]. Because of the characteristic of the dentin, dental bonding agents are now characterized as being hydrophilic to wetting dentin [76]. Dental bonding agent through mechanical and chemical combination improve the retention of the fillings in the nest inside the hole, slight leaking occurred in the lower margin [77].

Dental bonding agents are widely used in dental treatment, which can improve the sealing quality between resin and dentin, prevent bonding failure and reduce allergic reactions [78]. Dental bonding agents can be divided into two main categories: total-etch and self-etch. In total-etch bonding systems, acidic etchant composed of 35~37% of phosphoric acid can completely remove the dirt at the dentin surface and form a 3~5 µm loose network layer [79]. The bonding agent can infiltrate the collagen fiber network frame, forming a hybrid layer as the inter-lock. In self-etch bonding systems, unsaturated or polymerizable organic acid or an acid group as a functional component of the monomer methacrylic acid resin can play the role of etchant [80]. When acidic functional components and coupling agents were mixed together, tooth surface demineralization and coupling occurred simultaneously [81]. In this case, the independent acid etching step was omitted. Tooth bonding is a complex physical and chemical process [82,83].

### 3.1. Rheological Properties before Curing

Based on the bonding mechanism, a dental bonding agent is required to be in low viscosity before curing in order to better penetrate into the dentin tubules [84]. The usage of different monomers may affect the viscosity of dental bonding agent. Many adhesives today use Bis-GMA as the base resin, but the singular Bis-GMA is highly viscous and requires dilution with low viscosity dimethacrylate ether. The dimethacrylate ether has adverse effects on the curing and shrinkage properties of the bonding agent. Therefore, the viscosity of dental bond agent is usually reduced by chemical structural modification of Bis-GMA [85]. Kim et al. [86] found that the preparation of 2,2-Bis [4-(2-methoxy-3-methylacrylloxy propoxy) phenylpropane (Bis-M-GMA) by substitution of hydroxyl with methyl group in Bis-GMA can reduce the viscosity from 574 to 3.7 Pa·s, which further decreased the viscosity of dental bonding agent prepared with Bis-M-GMA. Jeon et al. [87] found a novel organic monomer, in which the alkoxy group was substituted for the hydroxyl group in Bis-GMA. The viscosity of the bonding agent decreased with the increase of the number of substituted monomers. In addition, environmental factors may also affect the viscosity of dental bonding agents. The bonding resin was usually stored in the refrigerator for chemical stability. Moraes et al. [88] found that the viscosity of the adhesive resin decreased with the increase of refrigeration time, and the bonding agent was recommended to be removed from the refrigerator at least 20 min before using.

Solvent is another important component of the adhesive, which can reduce the viscosity of the adhesive [89]. Wang et al. [90] studied the influence of different concentrations of ethanol solvent on the adhesive, and the result showed that the adhesive had lower viscosity and higher permeability when the ethanol content was at 30%. However, when the ethanol content reached 50%, the permeation rate of adhesive monomer decreased because too much ethanol diluted the ingredients [91]. Aw et al. [92] tested three kinds of adhesives, in which water, ethanol and none are used as solvents. After storing the bonded samples for a year, they found that all three types of bonded samples were able to maintain the original bond strength without significant differences.

Fillers can improve the elastic modulus of dental adhesives. Habib et al. [93] prepared the adhesives with Bis-GMA and UDMA resins and fillers of 75 nm, 150 nm, 350 nm, 500 nm and 1000 nm and measured the rheological properties of the adhesives with a rheometer. The unfilled adhesives had viscosities of 0.27–0.76 Pa·s, while the filled adhesives showed much higher viscosity when loaded at 70 wt%. Both the loading percent and particle size were considered as important reasons for this. Nanofillers have been very popular for the modifications of dental adhesives recently. Hydroxyapatite nanorods, as a new filler, can improve the elastic modules of dental boding agent [94]. She et al. [95] added polyacrylic acid and nano-clay into the adhesive to adjust the storage modulus of the adhesive by changing its content. They found that the elastic modulus of adhesive increased with the increase of clay and acrylic acid, which can reach 3000 MPa. Zandinejad et al. [96] prepared a new adhesive by mixing the alkane saline treated filler with monomer, and the penetration of polymeric monomer into the pores of the filling material significantly increased the elastic modulus.

### 3.2. Curing Properties

The proportion of monomer converted into polymer (degree of conversion, DC) is an important parameter to evaluate the cured content of dental adhesives. Phaneuf et al. [97] measured the DC of Adper Easy Bond (3M) cured by light emitting diode (LED), which was 72.8%. Moraes et al. [98] showed that the DC of Scotchbond Multi-Purpose (3M) was 59.3%. Zhang et al. [99] added 0, 1, 3, 5 and 7 wt% hydroxyapatite (HAP) to Adper prompt L-Pop (3M) and found that the DC increased from 7.8% to 58.4%. Barcelos et al. [100] added a new photoinitiator to the adhesive with the monomer of 2-hydroxyethyl methacrylate, and the DC of the new adhesive can reach as high as 86.2%. Ferreira et al. [101] used Ta_2_O_5_ to the adhesive containing methacrylate monomers and photoinitiators. The DC of Ta_2_O_5_ was 61.78%~67.35% in the range of 1~10 wt%. Watts et al. [102] studied the influence of the residual unevaporated ethanol on the DC of the polymer resin, and an adverse result was obtained with the group with residual ethanol. Eskandarizadeh et al. [103] modified the dental bonding agent with zinc dimethylate (ZDMA) and found that the DC of the bonding agent increased with the increase of ZDMA. In summary, the incorporation of new monomers, photoinitiator, filler and solvent may affect the DC of dental bonding agent to some extent, and a favorable DC is between 58% and 73% for clinical applications.

### 3.3. Mechanical Properties after Curing

The micro-tensile bond strength is typically measured by placing the bonded sample in water at room temperature for 24 h and holding the sample symmetrically on the gripper. When the distance from the clamp to the lap end is (50 ± 1) mm, the test machine is loaded at a steady speed of (5 ± 1) mm/min [104]. Fu et al. [105] measured the micro-tensile shear strength of a single step adhesives in the blowing time of 5–30 s. When the blowing time is between 15 and 30 s, the micro-tensile shear strength is higher. Fontes et al. [106] modified the dental adhesive with Tetrahydrofuran and compared the bond strength with the common adhesive. They found that the modified sub adhesive had a higher micro-tensile shear strength, which was 55.3 MPa. Inoue et al. [107] showed that the micro-tensile strength of a two-step total-etch adhesive Scotchbond 1 was 43.9 MPa. Jain et al. [108] obtained that the micro-tensile strength of the Optibond-FL adhesive system under water storage for six months was 49.69 MPa. The micro-tensile shear strength of dental adhesives is between 43 and 55 MPa. 

The shear bond strength is performed by placing a bonded specimen into a double shear pin joint and marking two shear positions, applying two shear forces at both ends of the joint until the specimen is fractured. Barkmeier et al. [93] tested the shear strength of Scotchbond Multi-Purpose (3M) with human enamel, and their obtained shear bond strength was between 13.4 and 21.9 MPa. Koh et al. [109] showed a single bottle of dental average adhesive strength of 21.3 MPa. Manuja et al. [110] compared the shear bond strength of Xeno III (Dentsply) and Adper Easy One (3M), and they found that Adper Easy One has a higher shear bond strength than Xeno III. The average shear bond strengths of Xeno III and Adper Easy One are 14.51 MPa and 23.68 MPa, respectively. Jamadar et al. [111] studied the shear bond strength of dental adhesives with different pH values and found that pH had no significant effect on the shear bond strength of the adhesives. Mustafa et al. [41] used 2-hydroxyethyl methacrylate (HEMA) in different proportion to modify dental adhesive. When the content of HEMA was 25 vol%, it had the highest shear bond strength of 21.15 MPa. In a nutshell, the shear bond strength is usually between 13 and 23 MPa, which is slightly lower than the results obtained with the micro-tensile method. Zhang et al. [112] proposed a new strategic structure: they developed an elastic layer material to prevent microleakage and water seepage of resin composites, providing us with an idea to shift from improving the mechanical properties of dental resin composites to a limited recovery life based on the use of an elastic layer between the composite binder and the dental tissue.

Over time, dental adhesives in the oral cavity are affected by factors such as temperature and water, resulting in a decrease in shear bond strength [113]. Thermal cycling can simulate oral environment and reduce shear bond strength [114]. Bishara et al. [115] measured that the shear bond strength of cyanoacrylate adhesive decreased about 80% after 500 thermal cycles. Paschos et al. [116] added light curing sealant Pro Seal (Reliance Orthodontic Products) to dentate bonding agent, and the shear bonding strength was measured to increase after thermal cycling.

## 4. Resin Luting Cement

At present, dental cements can be categorized to temporary and permanent depending on their clinical indications, which include five types by compositions: zinc phosphate, zinc polycarboxylate, glass ionomer, resin-modified glass ionomer and resin [117]. Resin luting cement mainly contains methacrylates (e.g., Bis-GMA, TEGDMA, UDMA), which is highly resembled to flowable dental composite [118]. Resin luting cement is functionalized to server as luting agent forming a connection between the indirect restorative material (e.g., crowns, veneers, inlays and onlays) and the repairable tooth structure, which is more resistant to occlusal wear than direct composite repair and is able to achieve proper proximal contact and occlusal [119].

Sufficient adhesion between the prosthesis and the teeth is important to the success of indirect restorations, where suitable resin luting cement must be properly selected. In addition, several color options are provided to meet the patient’s aesthetic requirements. Based on the clinical circumstances, resin cement is selected to be used as light-cured, dual cured and self-cured [120]. According to the application steps, resin luting cements can be divided into three types: etch and rinse, self-etch and self-adhesive. Self-adhesive resin luting cement can be used without a separate pretreatment or another dental adhesive [121], which is easy to use and greatly simplified the procedures.

### 4.1. Rheological Properties before Curing

Resin luting cements show viscoelastic behavior right after dispensing [122]. The rheological properties of the material depend on the type and concentration of the composition, and the presence of a large number of polymer substances will increase the flow resistance, leading to an increased apparent viscosity of the system. The viscosity of resin luting cement can be easily adjusted by changing the ratio of the resin matrix and filler particles [123]. Marcondes et al. [124] compared the viscosity of a flowable resin composite, Opallis Flow (FCG), two resin luting cements, RelyX Veneer (3M ESPE) and Variolink Esthetic LC (Ivoclar Vivadent), and they found that the viscosity of the flowable resin composite was smaller than those of the resin luting cement at the same temperature. When the temperature was set at 69 °C in their experiment, the viscosity of flowable composite was about 120–140 Pa·s, while the viscosity of resin luting cement ranged from 220 Pa·s to 1010 Pa·s. Sato et al. [125] found that the incorporation of acidic functional monomer (10-MDP) may increase the viscoelastic creep behavior of resin luting cement because of the reduction in the crosslinking polymer network, and the elastic modulus was 7.25–9.79 GPa in this case.

Different viscoelastic behavior of resin luting cements allows for their use in different clinical situations, where small film thickness and less polymerization stress can be typically correlated with low viscosity, which can effectively reduce the formation of crack and premature edge penetration [123]. Hahn et al. [126] suggested that resin luting cement with relatively higher viscosity can be used to reduce the microleakage of ceramic inserts. Overall, the resin-based dental cement exhibited similar rheological properties to flowable dental composites because of their resembled compositions and manipulating requirements despite of different restorative procedures.

### 4.2. Curing Properties

According to the methods of curing as mentioned previously, they can be divided into self-curing (or chemical-curing), light-curing and dual-curing [117]. The advantage of dual-curing is that it is capable of both self-curing and light-curing, which allows the cement to react when the strength of light is weak especially during the indirect restorations [122]. 

Yoshikawa et al. [127] found that a slow curing method can significantly reduce the polymer stress. The curing rate of light-cured cement is usually fast, which also produces greater polymerization stress than self-cured [127]. Bacchi et al. [128] demonstrated that thio-urethane oligomers can significantly reduce the polymerization shrinkage stress of light-curing resin cement. Their work [129] also showed that the addition of thio-urethanes to BisGMA-UDMA-TEGDMA (5:3:2, with 25 wt% silanated inorganic fillers) resin luting cement can reduce the polymerization stress without affecting the polymerization conversion rate. Turkoglu et al. [130] proved that the polymerization efficiency for resin cement cured under anterior monolithic zirconia may be superior to that cured beneath posterior monolithic zirconia. Lopes et al. [131] showed that the elastic modulus of double-cured resin cement is higher than that of chemically cured resin cement, which may be related to how they are cured.

Properly controlling the curing rate is important because an adequate operating time is required during the cementation. Faria-E-Silva et al. [132] studied the properties of BisGMA/TEGDMA-based dual-cured cement containing thiourethane (TU) and low concentrations of p-tolyldiethanolamnie (DHEPT) and benzoyl peroxide (BPO) as chemical initiators. They found that adding TU and using low concentrations of DHEPT/BPO increased the working time and reduced the polymerization stress. Moreover, the conversion rate was increased even without light. In addition to the curing method and illumination factors, the conversion of monomers within resin luting cement is also affected by the thickness of oxygen inhibition layer [12]. Differentiated from dental composite or dental bonding agent, it is not true for resin luting cement that the faster the curing, the better. The curing method, component ratios, exposure to oxygen and other additives or measurements should be considered to master a proper setting time in order to have an ideal time window for cementation.

### 4.3. Mechanical Properties after Curing

The mechanical properties of resin luting cement are similar to those of dental composites and dental bonding agenst, which strongly depend on the curing methods [133]. The mechanical properties after curing are usually determined by sliding contact, uniaxial compression and three-point bending experiments [134,135]. Some mechanical strength data of dental cement are summarized in Table 2, and it was illustrated that resin luting cement overall exhibited better tensile strength, toughness and extremely low solubility than other types of dental cements [136], which is strongly correlated to the introduction of dimethacrylate which allows the polymerization of the resin cement into dense crosslinked polymers with strong moisture resistance and durability [137].

Furthermore, the dual-curing of self-adhesive resin cement may enhance the bonding properties. Nagasawa et al. [139] studied the effects of sandblasting, hydrofluoric acid etching and primer on the shear bond strength of a self-adhesive resin cement between seven different CAD/CAM resin composites and one resin composite core, and it was shown that sandblasting or high frequency etching can effectively improve the bond strength of CAD/CAM blocks to dentin. The shear bond strength of the self-adhesive resin cement is 6.0–9.0 MPa after sandblasting, while it can reach 9.0–12.0 MPa after high frequency etching. The usage of primers can also improve the bonding effect of the adhesive resin cement and zirconium oxide. Maeda et al. [140] measured the shear bond strength (SBS) of zirconia ceramics with different primers and resin cement, and they found that the primer can significantly improve the shear bond strength of resin luting cement and zirconia ceramics to 9.0 to 20.8 MPa. Ahn et al. [137] studied the effects of different phosphate-containing monomer primers on the shear bond strength between MDP-containing self-adhesive resin cement and Yttria-tetragonal zirconia polycrystal (Y-TZP) ceramics, and they found that the highest bond strength (9.84–15.23 MPa) was obtained by combining an air grinding treatment and MDP-containing self-adhesive resin cement. Wolfart et al. [141] also showed that the use of MDP-containing self-adhesive resin cement on air-abraded zirconia ceramic can be recommended as a promising bonding method. Different pretreatment of the repair body surface also affects the bond strength between the resin luting cement and the repair body. Oezcan et al. [142] suggested that the bond strength of Bis-GMA-based luting cement to acid eroded glass ceramics (26.4–29.4 MPa) could be improved under dry conditions, and the silanized silica coating can significantly improve the bond strength of high aluminum ceramics with Bis-GMA-based luting cement. Moosavi et al. [143] suggested that the addition of water on the dentin surface does not promote the adhesion of self-adhesive resin cement to dentin.

Since there have been too many choices of dental cement products in the market, dentists are encouraged to make decisions based on different clinical circumstances. It seems that the resin-based cements are playing a more and more important role attributing to their mechanical advantages and simplicity in decision making when selecting suitable products.

## 5. Conclusions and Future Perspective

The rheological and mechanical properties of resin-based dental materials not only determine their handling properties, but also affect their service life after clinical use. Dental filling composites can be divided into universal composites and flowable composites based on their consistency, which have been widely used for dental restorations. The rheological properties of universal composite resins are related to filler volume, filler particle diameter and temperature. In addition, universal composites can be modified by changing the type or amount of filler and the resin matrix. Compared with the general resin, the flowable composite filler has lower load, better flowability, weaker mechanical properties and lower viscosity. The flowable composite has the advantages of easy handling and good permeability, and its tooth edge has good adhesion, its shrinkage rate is less than that of the common composite material and the curing depth is more ideal. The content of filler is directly proportional to its viscosity, so it is an idea to modify the properties of dental composites. Dental adhesive bonds dental filling composite to tooth tissue and has a very low viscosity, which helps it to be coated. Compared with other resin-based dental materials used in direct restorations, resin luting cement used in indirect restorations has the advantages of various curing methods. In addition, the mechanical properties and rheological properties of resin-based dental cements were found to be superior to other types of dental cements and might be affected by the cement formulations and curing protocols.

Dental filling composites, dental adhesives and resin-based dental cements have both similarities and differences in their rheological and mechanical properties with different applications in dental restorations. With the development of technology and the improvement of materials, the resin-based materials are continually being optimized in the trend of becoming more convenient for clinical operations and more functional, playing an all-in-one role. 

## Figures and Tables

**Table 1:** Viscosity of monomers and different combinations for matrix of dental composite.

Monomer	Temperature (°C)	Viscosity (Pa·s)	Reference
Bis-GMA	24.1	426.6	[26]
Bis-GMA	25	369	[28]
Bis-GMA	35	52.6	[28]
Bis-GMA (80 wt%) + MMA (20 wt%)	24.1	0.66	[26]
Bis-EMA 10	20	0.5675	[29]
Bis-EMA 30	20	0.8213	[29]
UDMA	20	6.878	[29]
UDMA	23	7.054	[30]
HEMA	20	0.0149	[29]
UDMA (75 wt%) + HEMA (25 wt%)	23	0.365	[30]
UDMA (40 wt%) + HEMA (60 wt%)	23	0.021	[30]
TEGDMA	25	0.0077	[29]
TEGDMA	35	0.0068	[28]
UDMA (80 wt%) + TEGDMA (20 wt%)	23	0.655	[30]
UDMA (20 wt%) + TEGDMA (80 wt%)	23	0.048	[30]

Bis-GMA, bisphenol A glycidyl methacrylate; MMA, methyl methacrylate; Bis-EMA, ethoxylated bisphenol A glycol dimethacrylate; UDMA, urethane dimethacrylate; HEMA, 2-hydroxyethyl methacrylate; TEGDMA, triethylene glycol dimethacrylate.

**Table 2:** Mechanical properties of various luting cements.

	Setting Time (min)	Compressive Strength (MPa)	Tensile Strength (MPa)	Modulus of Elasticity (GPa)	Reference
Zinc Phosphate	5–9	96–133	3.1–4.5	13	[117]
Zinc Polycarboxylate	7–9	57–99	3.6–6.3	5–6	[117]
Glass-ionomer	6–8	93–226	4.2–5.3	7–8	[117]
Resin-modified Glass-ionomers	-	150–220	-	-	[138]
Resin	4+	180–265	34–37	4–6	[117]

## Data Availability

Not applicable.
